# Supplementary material for: Modulating inherent lewis acidity at the intergrowth interface of mortise-tenon zeolite catalyst
Source: Nat Commun. 2022 May 25;13:2924. doi: 10.1038/s41467-022-30538-7 (PMC9133034; doi:10.1038/s41467-022-30538-7)
Supplement: Supplementary file 1 — Supplementary Information [file 41467_2022_30538_MOESM1_ESM.pdf]

Supplementary Information

Modulating Inherent Lewis Acidity at the Intergrowth Interface of Mortise-Tenon Zeolite Catalyst

Huiqiu Wang[1#], Boyuan Shen[1,2#], Xiao Chen[1]*, Hao Xiong[1], Hongmei Wang[1], Wenlong Song[1], Chaojie Cui[1], Fei Wei[1]*, Weizhong Qian[1]*

Affiliations:

[1]Beijing Key Laboratory of Green Chemical Reaction Engineering and Technology, Department of Chemical Engineering, Tsinghua University, Beijing 100084, China.

[2]Institute of Functional Nano & Soft Materials (FUNSOM), Jiangsu Key Laboratory for Carbon-Based Functional Materials & Devices, Soochow University, 199 Ren'ai Road, Suzhou, 215123, Jiangsu, PR China.

[#]These authors contributed equally: Huiqiu Wang, Boyuan Shen.

*Corresponding author:

Email: chenx123@tsinghua.edu.cn (X.C.); wf-dce@tsinghua.edu.cn (F.W.); qianwz@tsinghua.edu.cn (W.Q.)

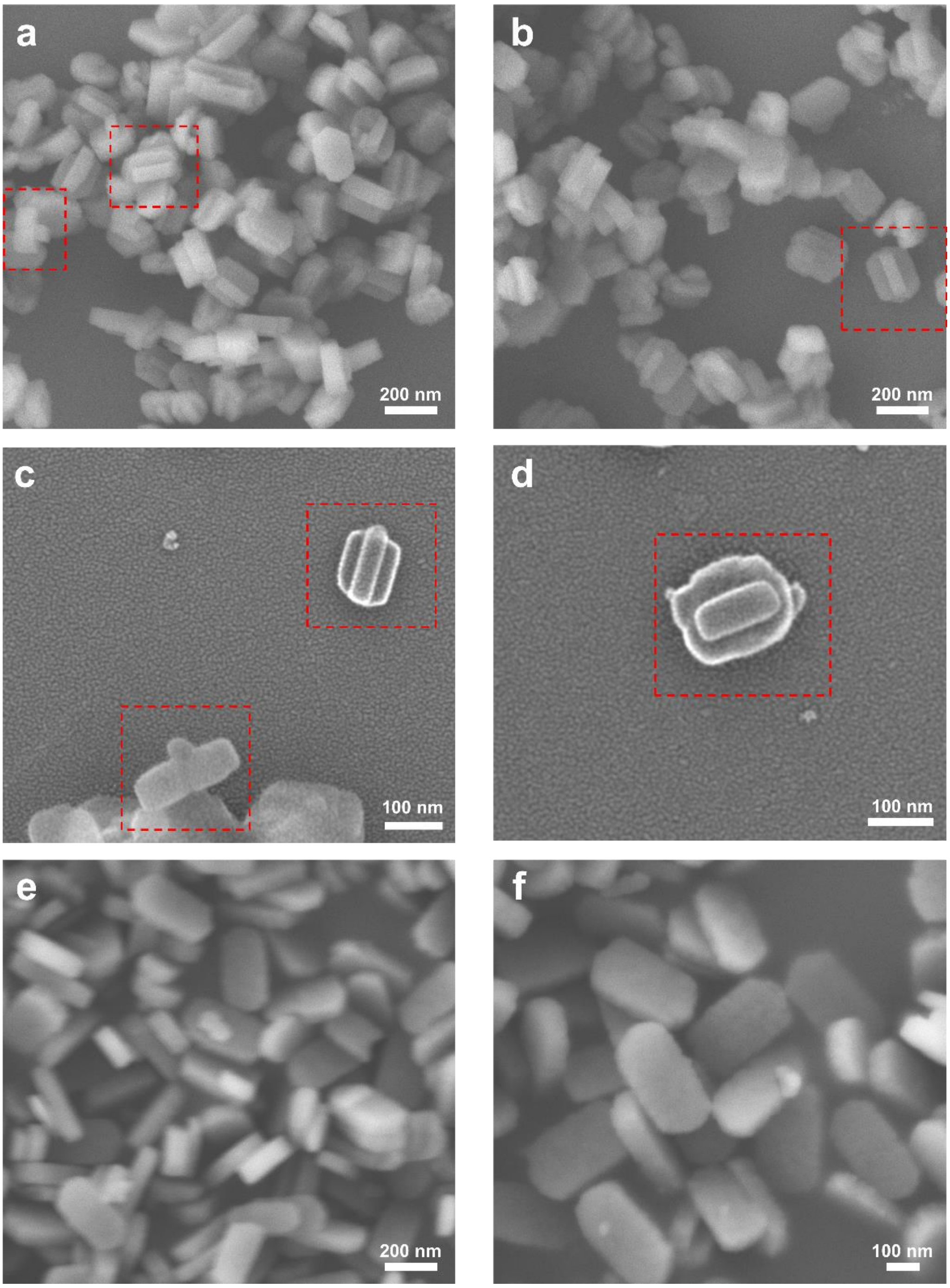

Supplementary Figure 1. Morphological characterization of zeolites. The SEM images of ZSM-5-MT (a, b, c, d) and ZSM-5-Sb (e, f) samples.

Note: The ZSM-5-MT nanocrystals exhibit a highly intergrowth morphology with a tenon-like protrusion vertically growing on the (010) surface of a underlying ZSM-5 crystal. From the lateral view, the fin-like protrusion and underlying crystal are closely attached without obvious gaps. While, the ZSM-5-Sb nanocrystals show a coffin shape with clean (010) surface.

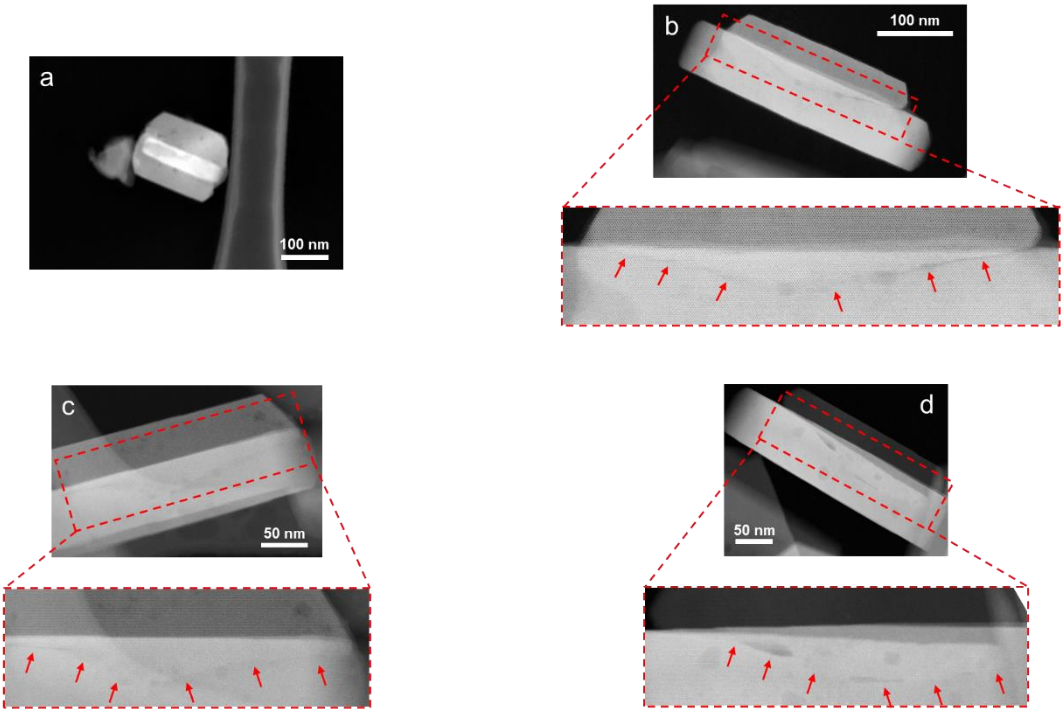

Supplementary Figure 2. More ADF-STEM images of ZSM-5-MT. (a-d) The images of some individual ZSM-5-MT crystals. The boundary between tenon and underlying mortise crystal marked by red arrows in the magnified image.

Note: In Supplementary Figure 2a, the projected morphology of individual ZSM-5-MT crystal is consistent with the results from the SEM images. More importantly, from the lateral view (Supplementary Figure 2b-d), a gray curve inside the underlying crystal represent the boundary between fin-like protrusion and underlying crystal, which due to the missing of O atoms at the intergrown interfaces. Thus, we can conclude that the tenon tongue physically enter the mortise hole and the mortise-tenon structural model can be solidly validated.

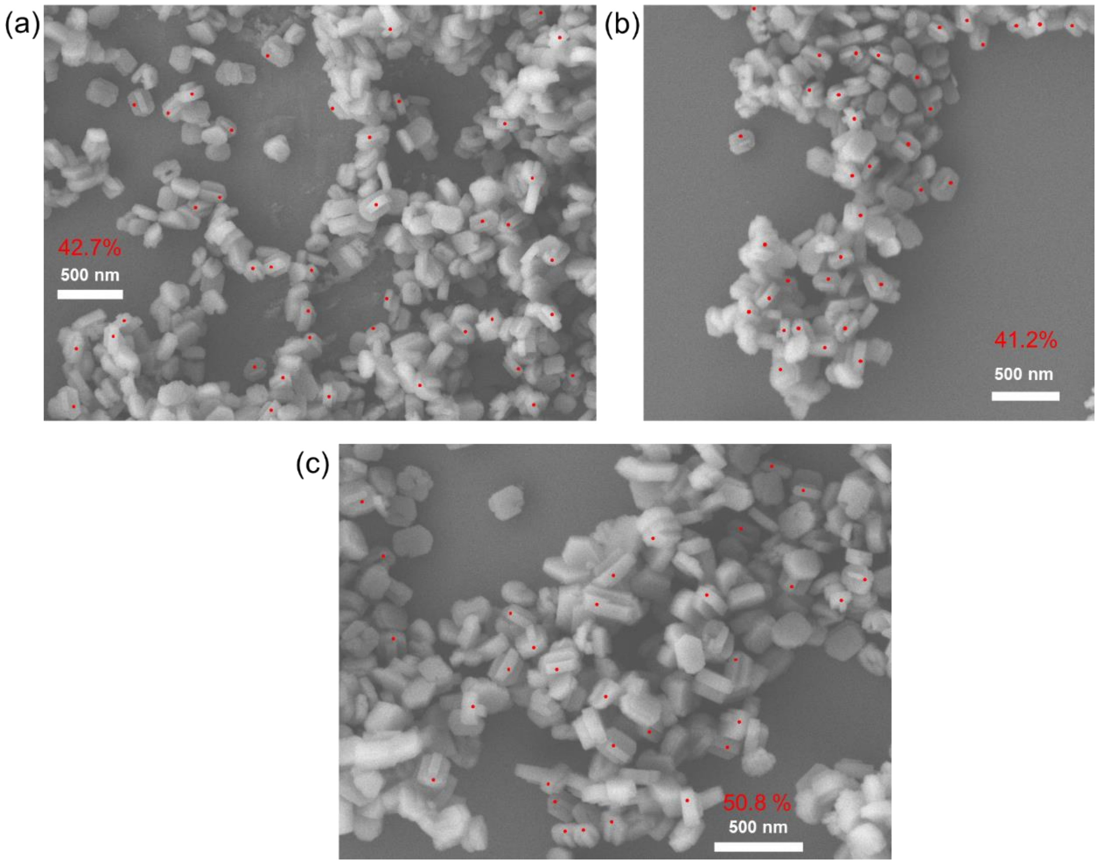

Supplementary Figure 3. The statistics of the yield of ZSM-5-MT crystals using SEM images. ZSM-5-MT crystals are marked by red dots. The yield of ZSM-5-MT crystals 42.7% (a), 41.2% (b), and 50.8% (c).

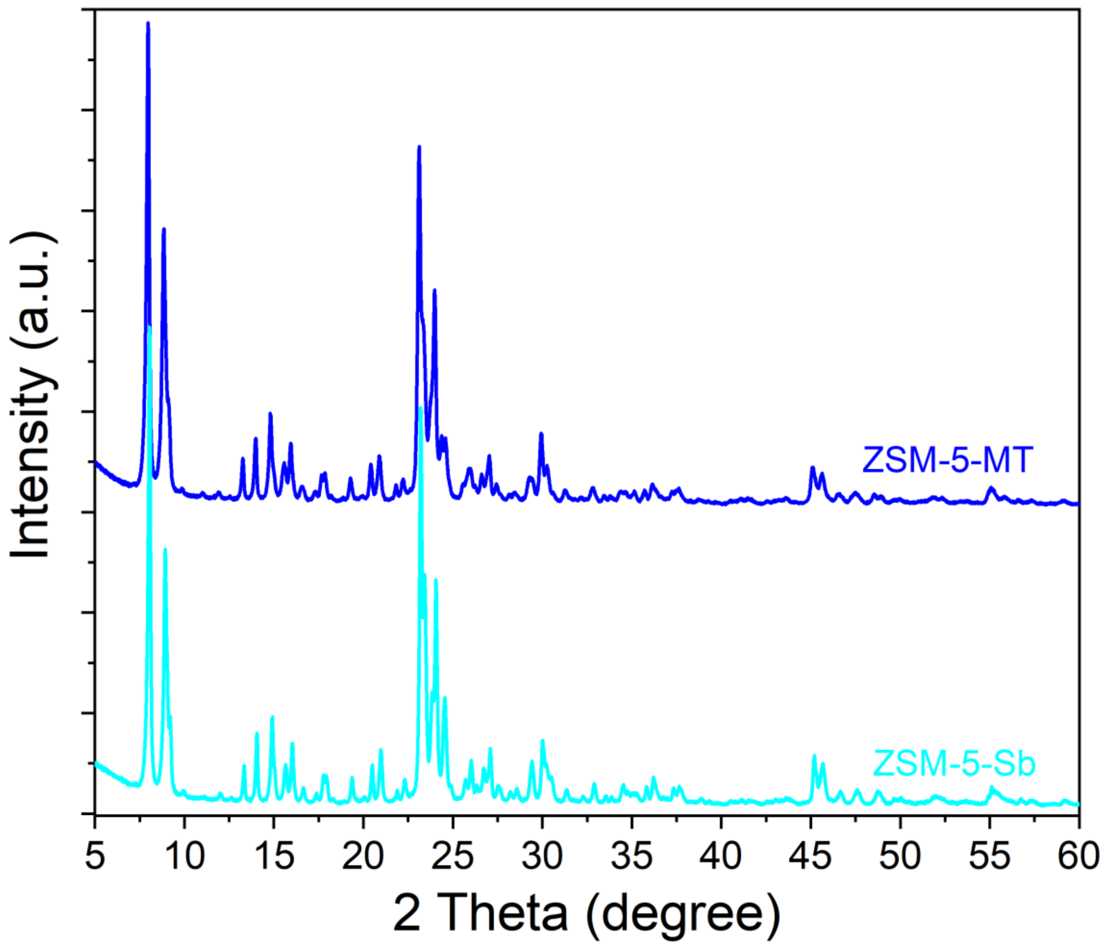

Supplementary Figure 4. XRD patterns for ZSM-5-MT and ZSM-5-Sb samples. Both ZSM-5-MT and ZSM-5-Sb show a pure MFI zeolite phase with excellent crystallinity.

Supplementary Table 1. Chemical, textural properties of the ZSM-5-MT and ZSM-5-Sb samples.

Sample name	Si/Al atomic ratio[a]	BET surface area (m^2/g)	Micropore surface area[b] (cm^3/g)	Total pore volume (cm^3/g)	Micropore volume (cm^3/g)	Mesopore volume[c] (cm^3/g)
ZSM-5-MT	61	416.7	374.8	0.243	0.136	0.107
ZSM-5-Sb	71	446.2	409.1	0.268	0.147	0.121

[a]Measured by ICP-OES.

[b]t-plot method.

[c]V_{meso}=V_{tot}-V_{micro}

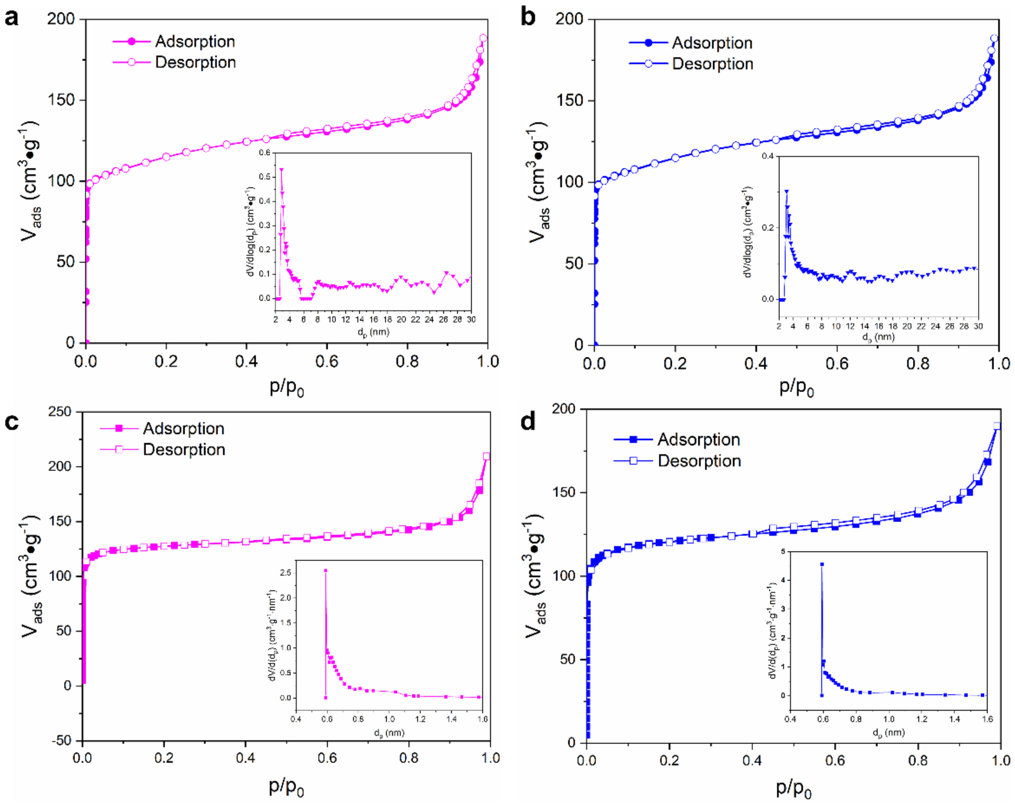

Supplementary Figure 5. Gas adsorption-desorption isotherms. N_2 adsorption-desorption isotherm for the ZSM-5-Sb (a) and ZSM-5-MT (b) samples. The insets are corresponding mesopore size distributions. Ar adsorption-desorption isotherms for the ZSM-5-Sb (c) and ZSM-5-MT (d) samples. The insets are corresponding micropore size distributions.

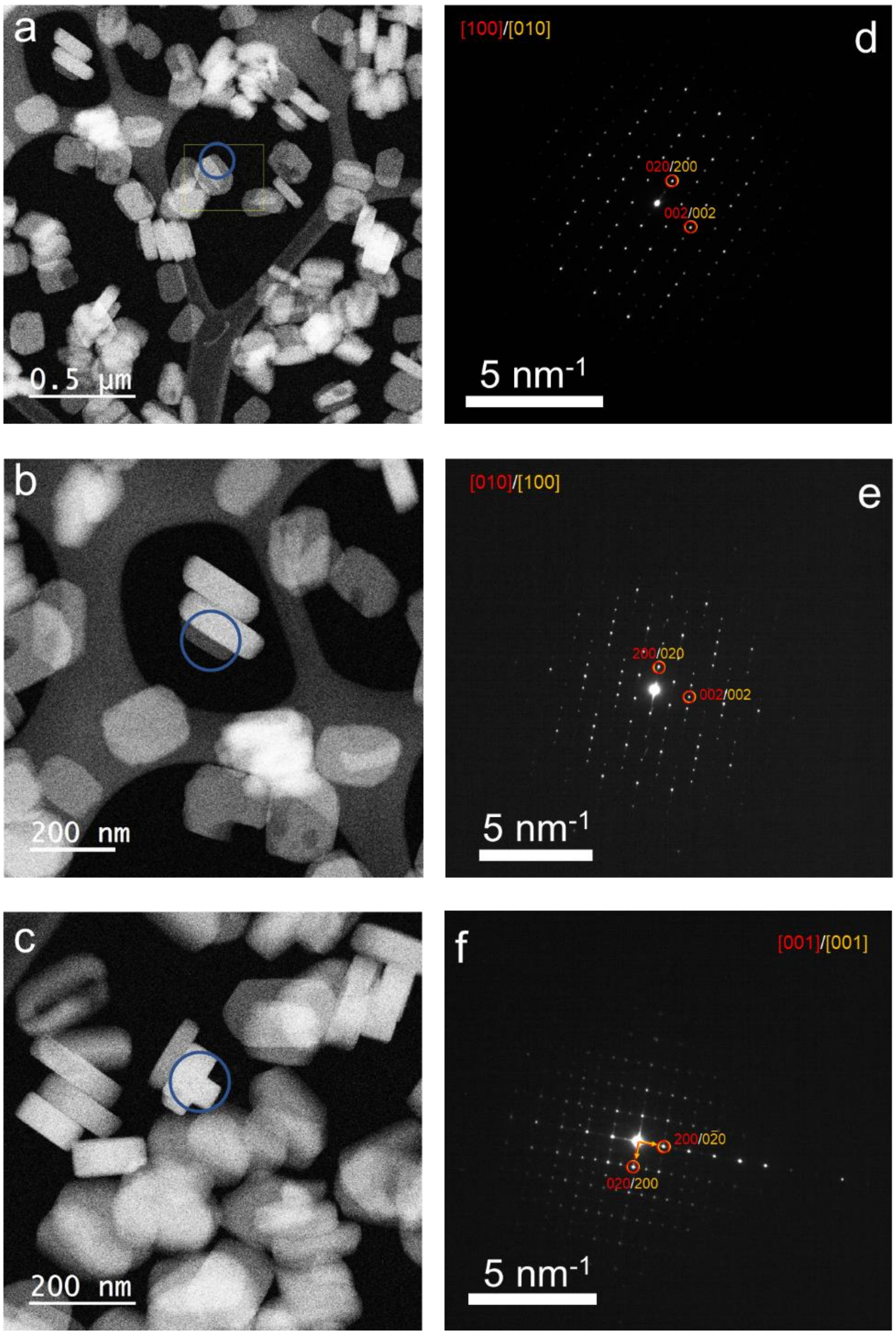

Supplementary Figure 6. The electron diffraction characterization of ZSM-5-MT. (a-c) The STEM images of ZSM-5-MT crystals along [100], [010] and [001] projections of tenon subunit, respectively. (d-f) The selected area electron diffraction (SAED) patterns correspond to the areas marked by blue circle in (a-c), respectively.

Note: The (010) and (100) surfaces of tenon subunit are perfectly connected with the (100) and (010) surfaces of mortise subunit, respectively. Simultaneously, the tenon subunit and mortise subunit share the common **c** axis

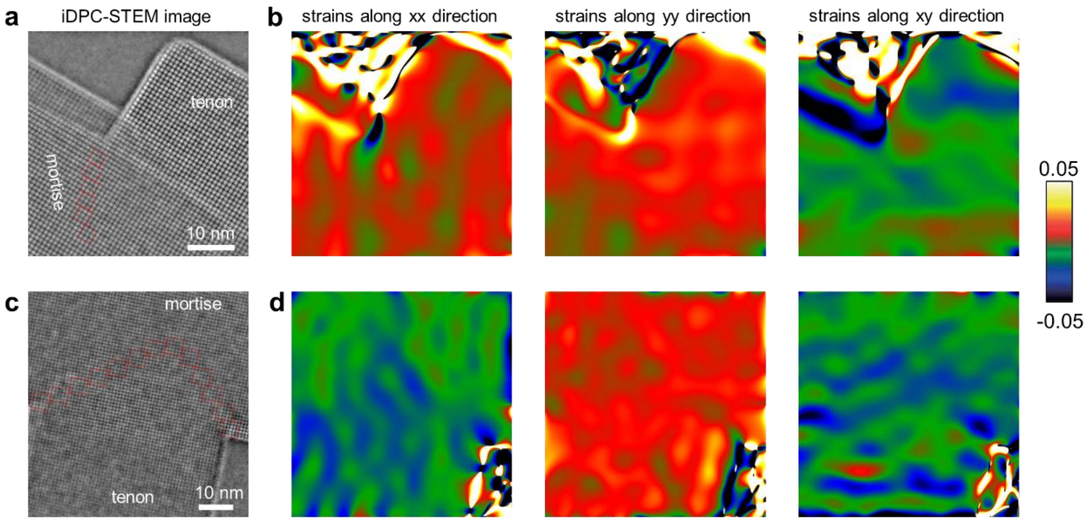

Supplementary Figure 7. Strain analysis of ZSM-5-MT crystals. (a and c) The iDPC-STEM images of ZSM-5-MT crystals from [001] direction. (b and d) The GPA strain distribution maps corresponding to (a) and (c), respectively. The interfaces are marked by red dotted frames in images.

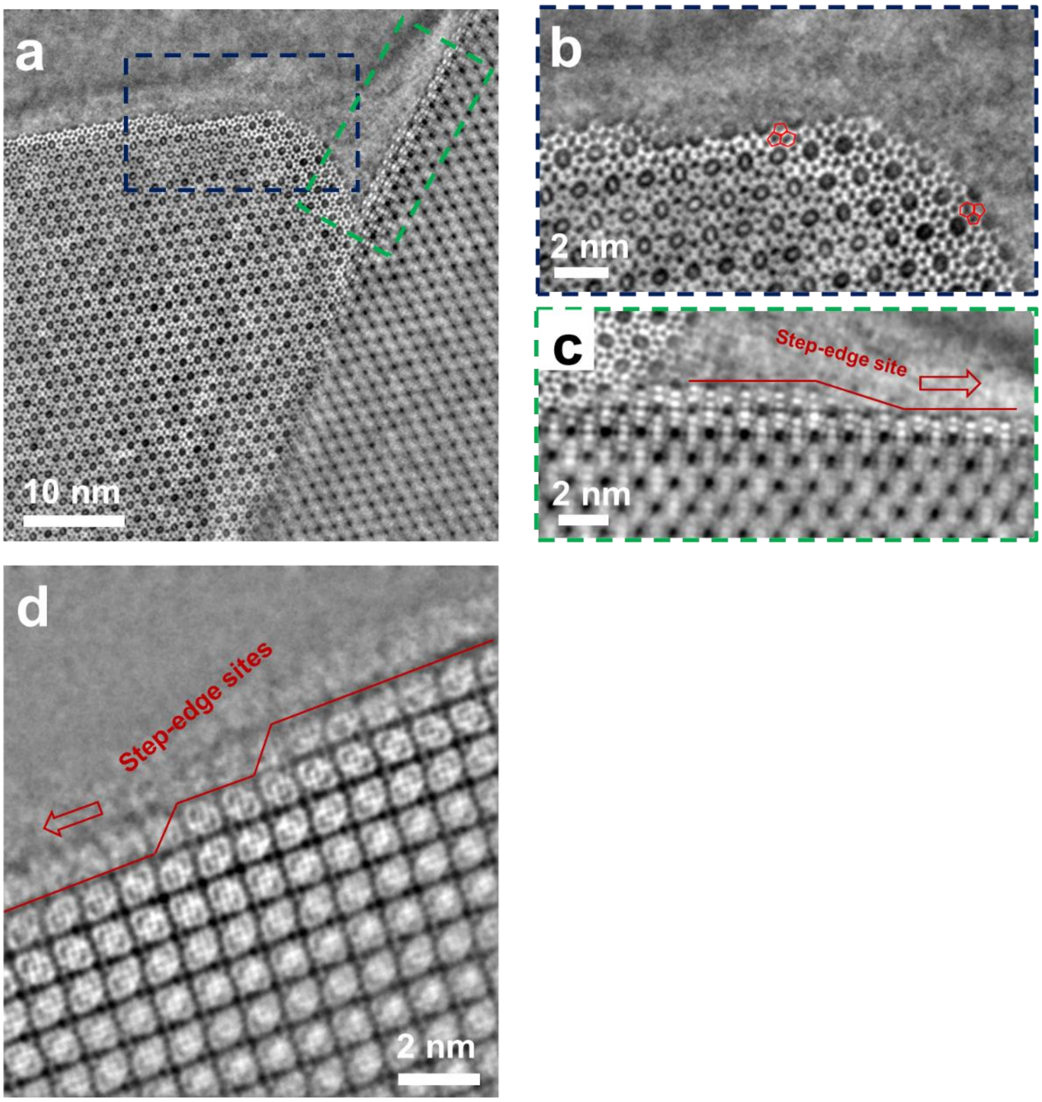

Supplementary Figure 8. Imaging the surface terminations by iDPC-STEM. (a) The surface terminations of mortise ZSM-5 crystal from the lateral direction. (b and c) The magnified iDPC-STEM images in the areas marked in (a). (d) The surface terminations of mortise ZSM-5 crystal from the [001] direction.

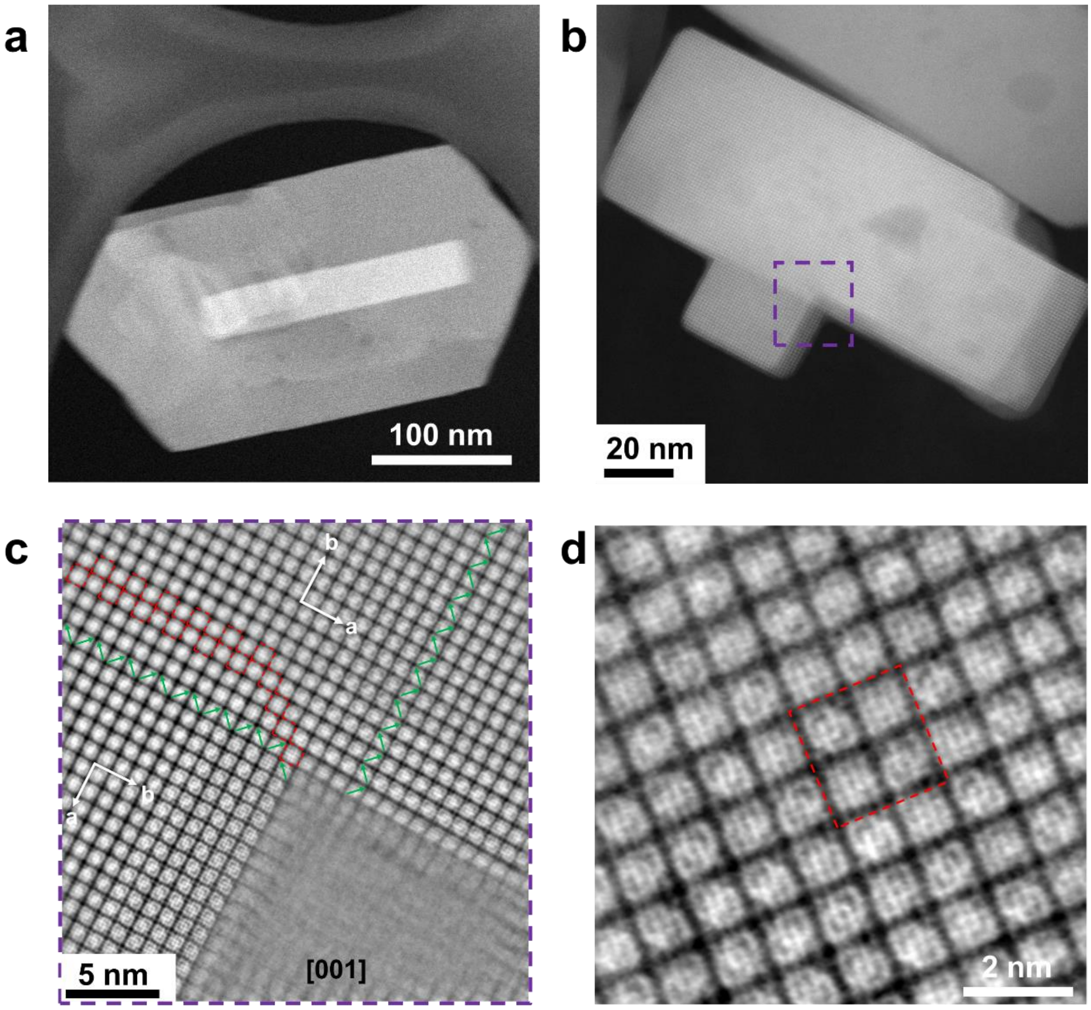

Supplementary Figure 9. More images about the interfacial analysis of ZSM-5-MT. (a, b) The ADF-STEM images of ZSM-5-MT nanocrystals. (c) The magnified iDPC-STEM image of the area marked as purple frame in (b). (d) The iDPC-STEM image of the intergrown area from the [001] direction. The magnified iDPC-STEM image Fig. 2 was obtained from the dash red frame in Supplementary Figure 9d.

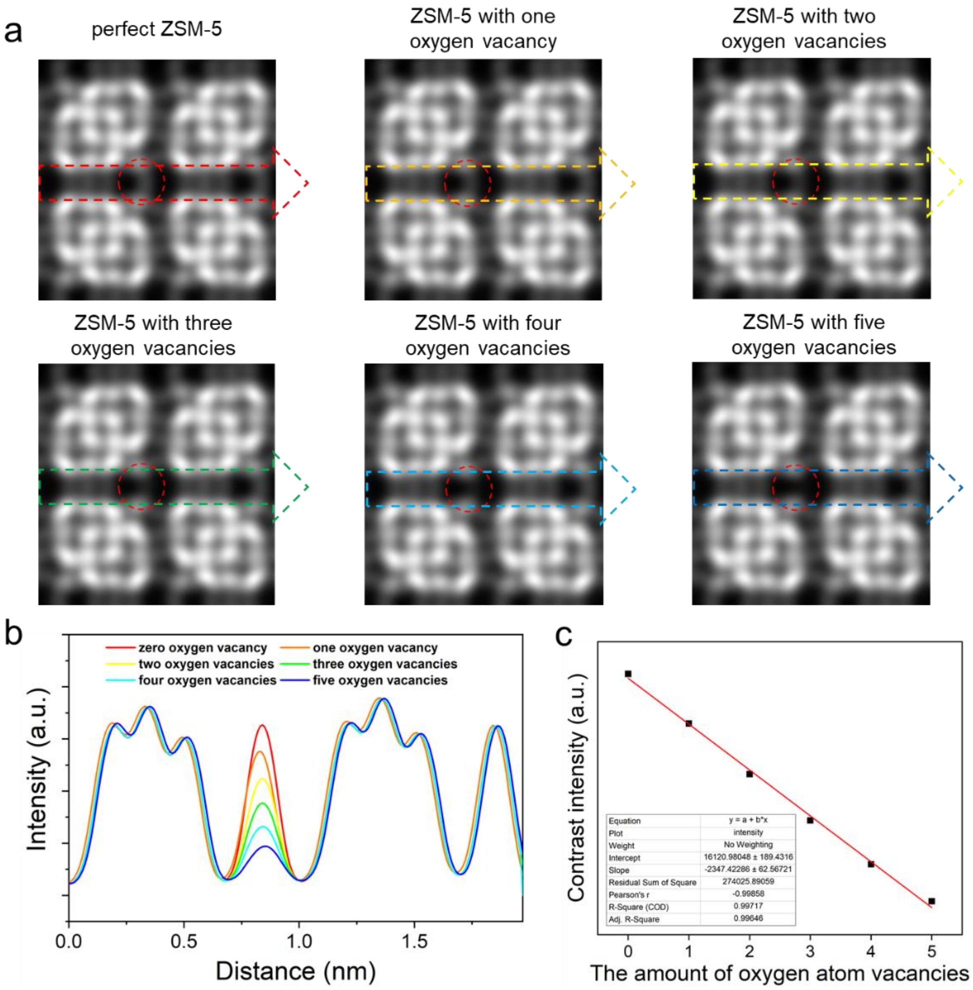

Supplementary Figure 10. The iDPC-STEM image simulations. (a) The simulated iDPC-STEM images of five ZSM-5 unit cells with the different amounts of oxygen vacancies (The oxygen atom columns with vacancies are marked by dash red circles). (b) The intensity profiles of six ZSM-5 frameworks (six color dash arrows shown in the top six images) with different amount of oxygen vacancies. (c) The contrast intensity of the oxygen atom columns related to the amount of oxygen vacancies.

Note: We performed iDPC-STEM image simulations to investigate the relation between the iDPC contrast and the amount of oxygen molecules. We give the simulated results of several different samples in Supplementary Figure 10a, including the perfect ZSM-5 and the defective ZSM-5 with different amount of oxygen vacancies. The intensity profiles indicate that the height of oxygen column peak is related to the amount of oxygen vacancies. Meanwhile, we can actually find that the contrast intensity of oxygen atom column is linearly related the amount of oxygen vacancies. Thus, the iDPC contrast of oxygen atom column is quantitative.

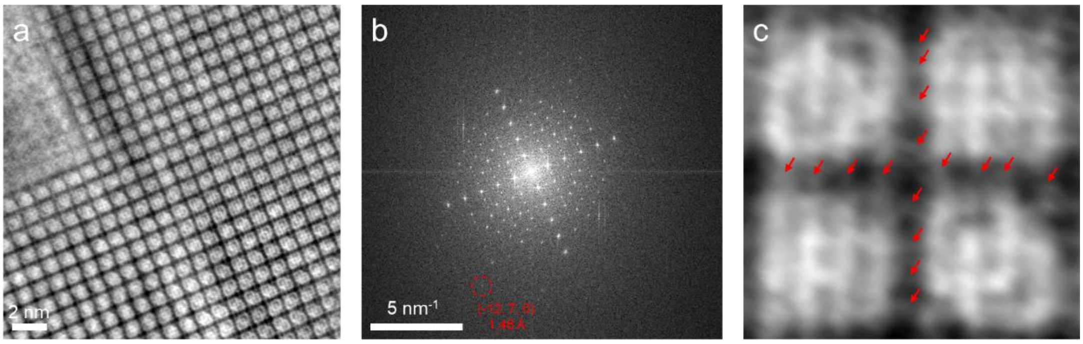

Supplementary Figure 11. The characterization of interfacial oxygen columns. (a) The iDPC-STEM image of ZSM-5-MT crystal from the [001] direction. (b) The corresponding FFT pattern of (a) in a log scale. (c) The magnified iDPC-STEM image of the intergrown area in (a) (individual oxygen columns are marked by red arrows).

Note: In the corresponding FFT pattern of Supplementary Figure 11a, the (-12,7,0) plane shows an information transfer of 1.46 Å, which is sufficient to resolve individual oxygen columns, as the distance between adjacent oxygen atoms is always larger than 1.46 Å. As expected, sixteen individual oxygen columns are clearly identified in the magnified image (see Supplementary Figure 11c).

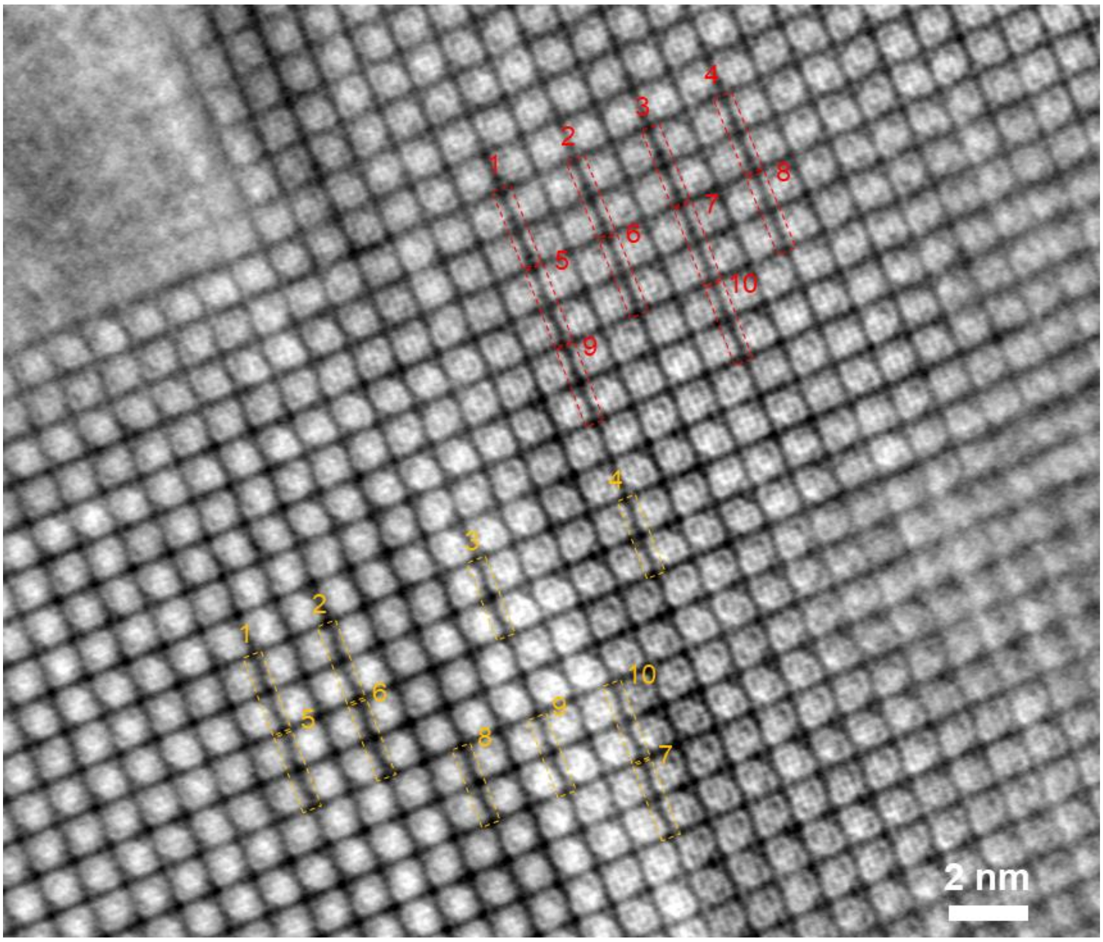

Supplementary Figure 12. The iDPC-STEM imaging of ZSM-5-MT crystal from the [001] projection. To statistically analysis the contrast of bridged O atom columns, we selected ten different sections in intergrowth areas (marked by red dash rectangle frame) and single-crystal areas (marked by yellow dash rectangle frame), respectively.

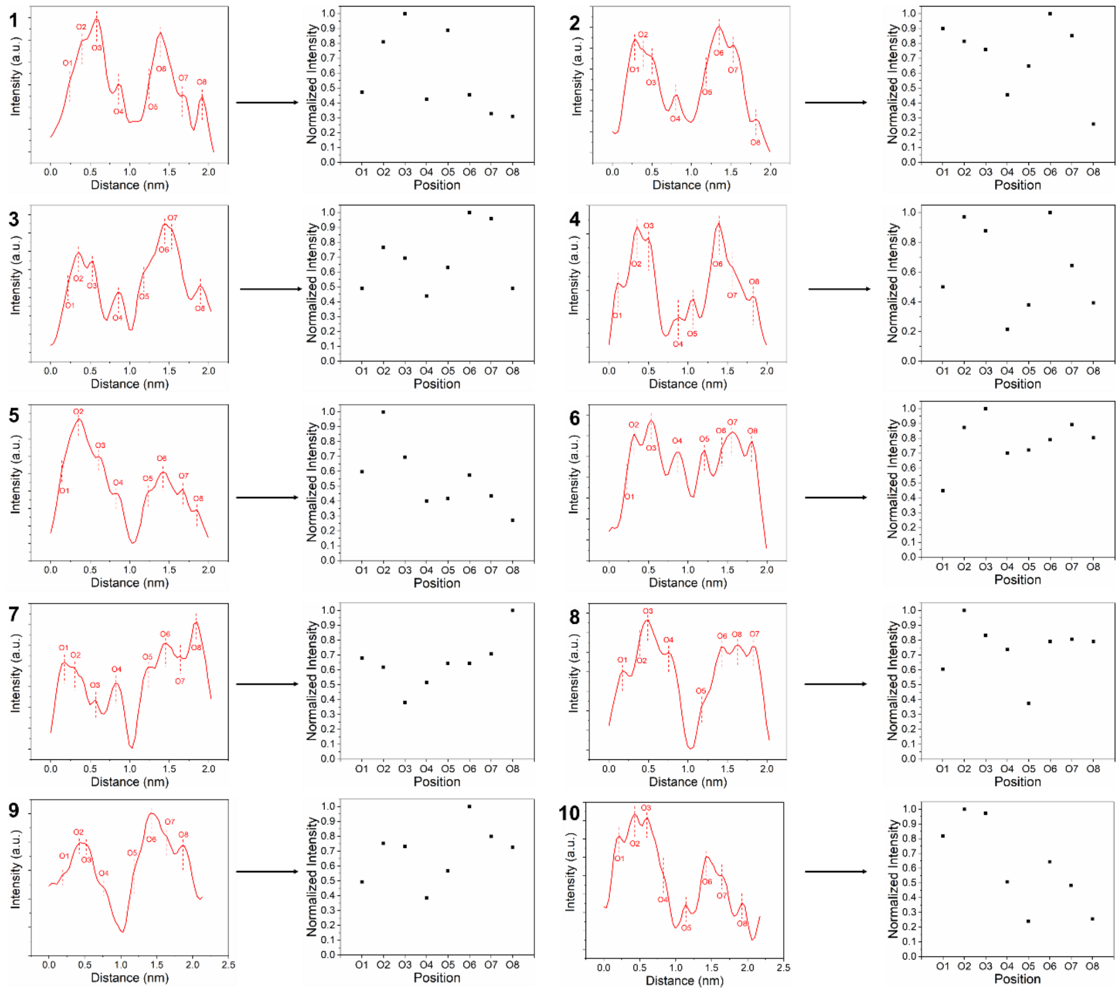

Supplementary Figure 13. The corresponding profile analysis as marked by red dash rectangle frames in Supplementary Figure 12.

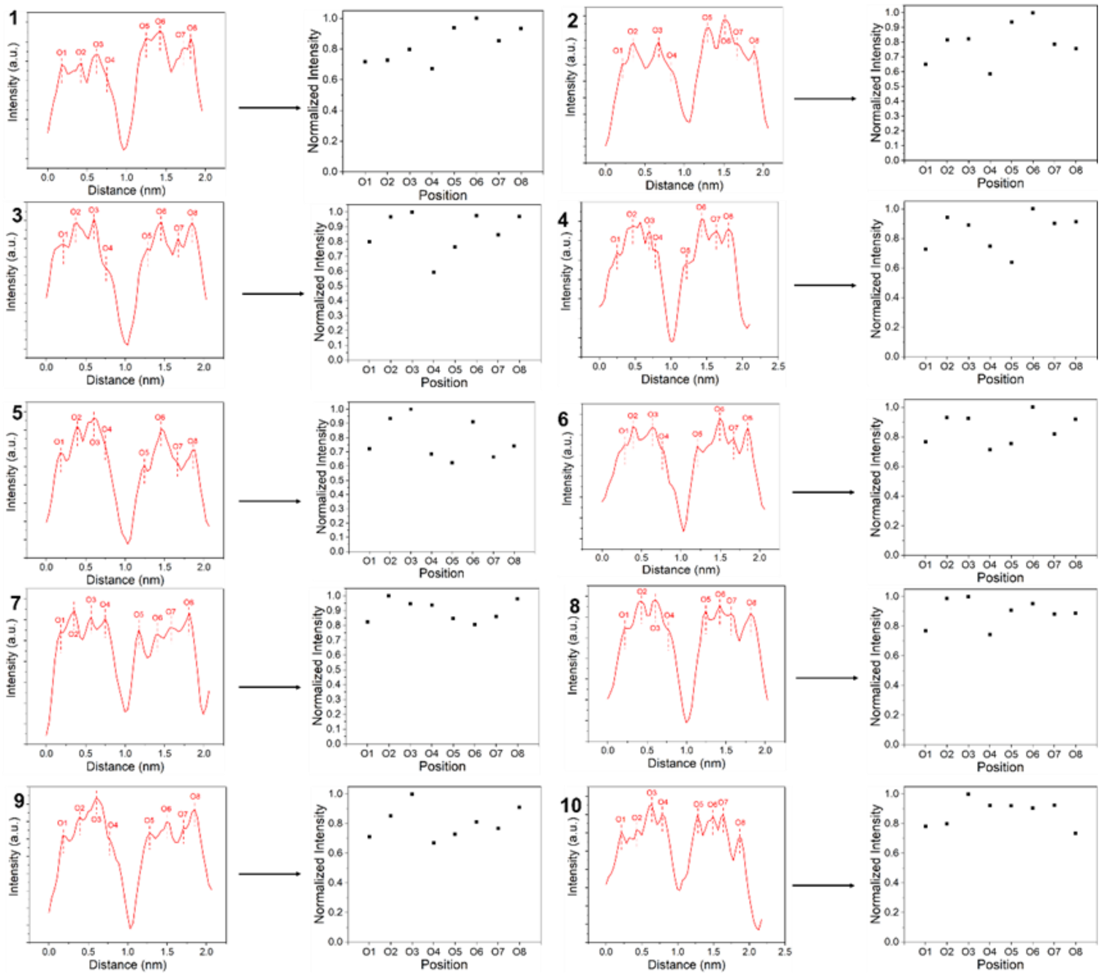

Supplementary Figure 14. The corresponding profile analysis as marked by yellow dash rectangle frames in Supplementary Figure 12.

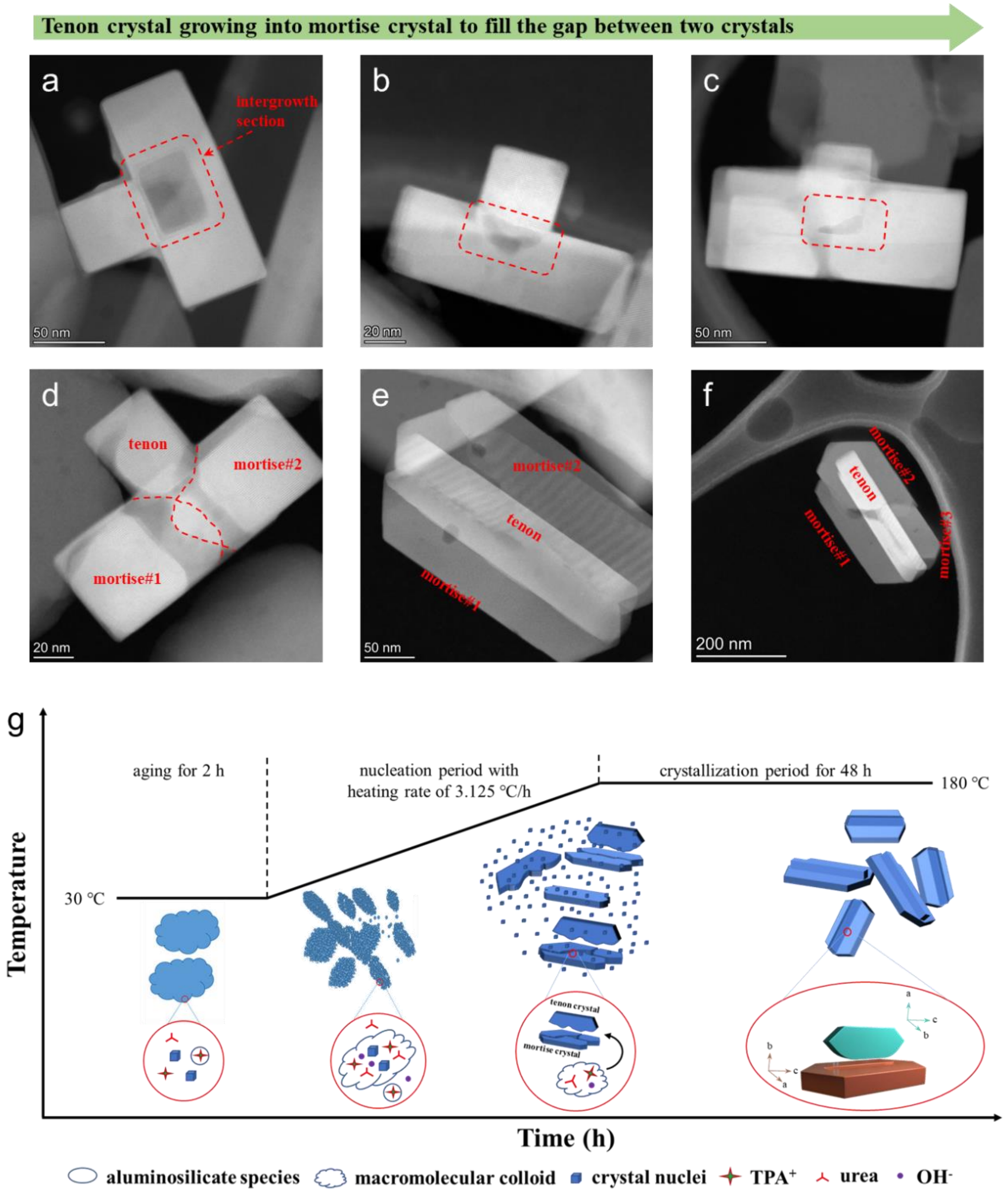

Supplementary Figure 15. Investigating the growth mechanism of ZSM-5-MT. ADF-STEM analyses of embryonic ZSM-5-MT crystals in different crystallization stages (a-f). Schematic Presentation of the system's evolution leading to ZSM-5-MT crystals (g). Note: To investigate the growth mechanism of ZSM-5-MT, we captured some embryonic ZSM-5-MT crystal without fully grown structure in the course of zeolite synthesis. In Supplementary Figure 15a-c, a gray hole with different size is observed between tenon and mortise subunit in each embryonic ZSM-5-MT crystal. Due to the fact that a higher growth rate along **a** axis as compared with the **b** axis, the tenon crystal

growing into mortise crystal to fill the gap between two crystals[1]. Furthermore, we found that the embryonic ZSM-5-MT crystals consist of more than two components (Supplementary Figure 15d-e). It indicates that more than two discrete zeolite crystals assembled into primary ZSM-5-MT crystal. Then, the primary ZSM-5-MT crystal was completely converted into ZSM-5-MT crystal through additional crystallization.

As mentioned in Methods, the heating rate of crystallization temperature and the amount of NaOH was respectively decreased and increased to obtain ZSM-5-MT crystals. Increasing alkalinity will speed up the crystallization of zeolites. And, we anticipate that slowing down the heating rate of crystallization temperature can increase the amount of crystal nuclei and discrete zeolite crystals[2]. As a result, the probability of forming intergrowth structure increased with an increase of the density of crystal nuclei and discrete zeolite crystals. On the basis of collected results and previous studies[3], the growth mechanism of ZSM-5-MT crystals is presented in Supplementary Figure 15g.

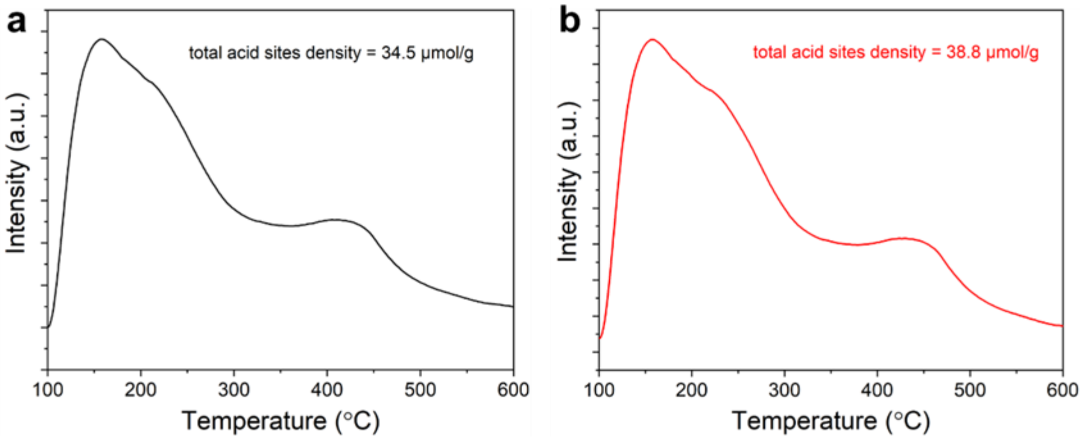

Supplementary Figure 16. Acidity characterization. NH3 temperature-programmed desorption (TPD) profiles for ZSM-5-MT (a) and ZSM-5-Sb (b). The acid density was normalized to the weight of sample.

Supplementary Table 2. The acid site concentrations determined from FT-IR spectra of adsorbed pyridine.

Sample	Acid site concentration (μmol/g)		ratio of BASs/LASs
	LAS	BAS	
ZSM-5-MT	14.22	167.63	11.79
ZSM-5-Sb-67	17.62	207.83	11.80
ZSM-5-Sb-75	14.80	143.30	9.68

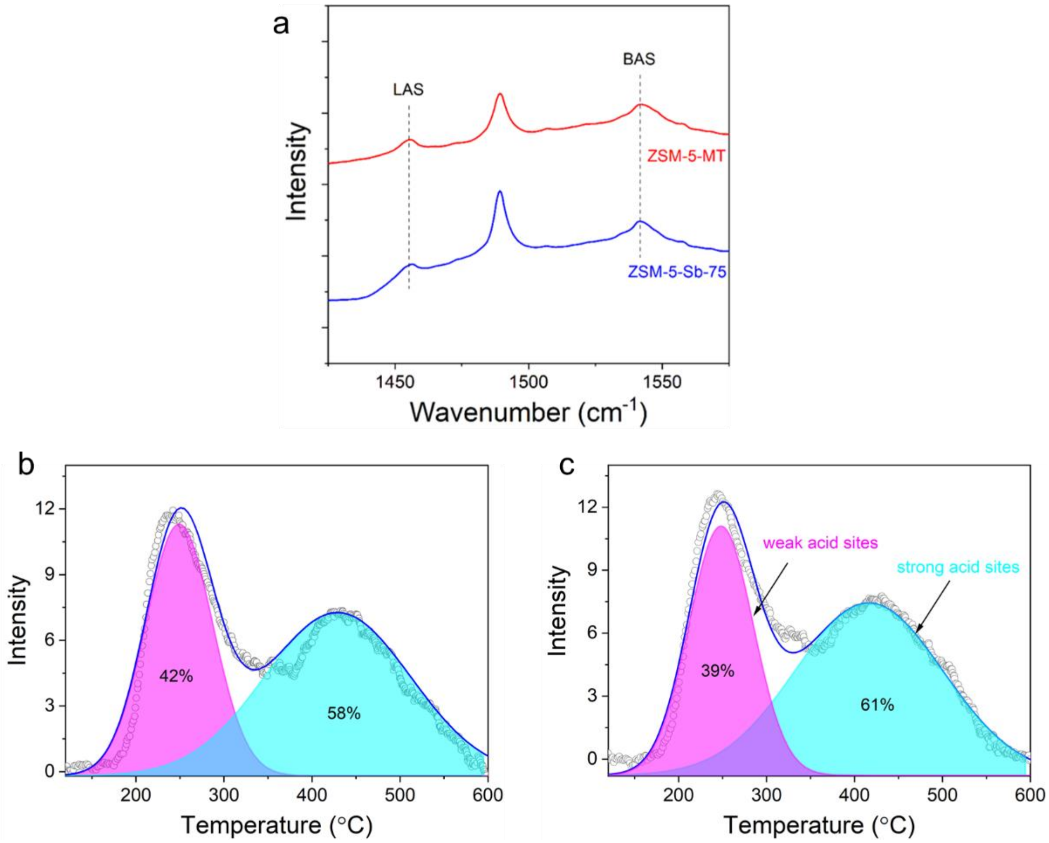

Supplementary Figure 17. Acidity properties comparison between ZSM-5-MT and ZSM-5-Sb-75. (a) FTIR spectroscopy of adsorbed pyridine in ZSM-5-MT and ZSM-5-Sb-75. NH_3 temperature-programmed desorption (TPD) profiles for ZSM-5-MT (b) and ZSM-5-Sb-75 (c).

Note: The BAS numbers and acid sites strength of these two catalysts are identical.

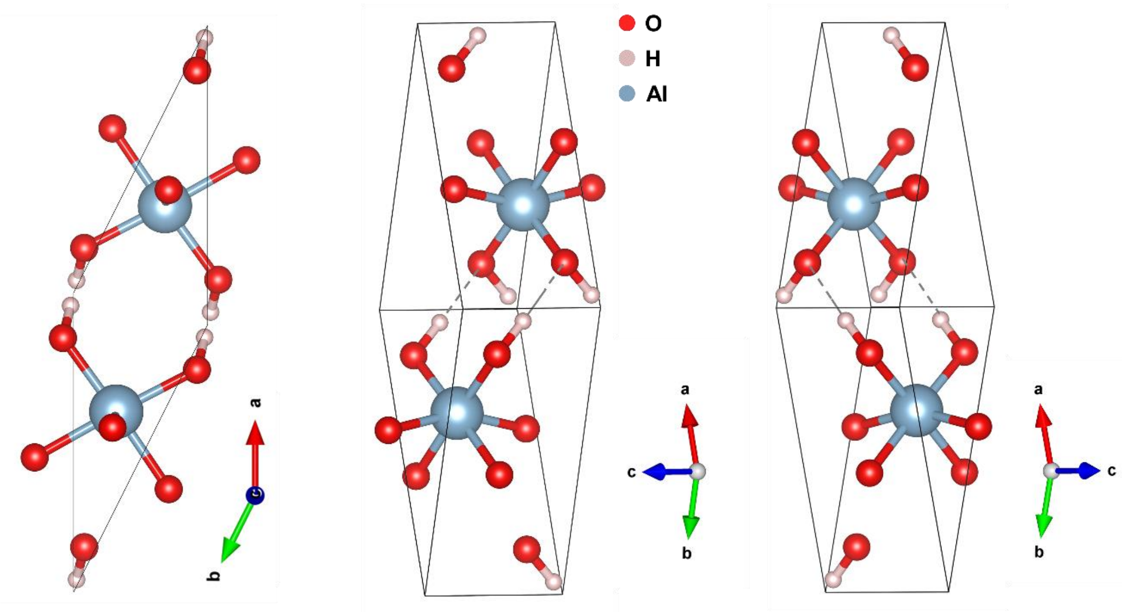

Supplementary Figure 18. The structure model of the boehmite (γ-AlOOH) from three different lattice directions.

Supplementary References

1. Karwacki L*, et al.* Morphology-dependent zeolite intergrowth structures leading to distinct internal and outer-surface molecular diffusion barriers. *Nat. Mater.* **8**, 959-965 (2009).

2. Xu R, Pang W, Yu J, Huo Q, Chen J. Chemistry of Zeolites and Related Porous Materials: Synthesis and Structure. (John Wiley & Sons, Singapore, 2007).

3. Dai W, *et al.* Platelike MFI Crystals with Controlled Crystal Faces Aspect Ratio. *J. Am. Chem. Soc.* **143**, 1993-2004 (2021).
